# Supplementary material for: Magnetism-induced topological transition in EuAs3
Source: Nat Commun. 2021 Nov 30;12:6970. doi: 10.1038/s41467-021-26482-7 (PMC8635340; doi:10.1038/s41467-021-26482-7)
Supplement: Supplementary file 1 — Supplementary Information [file 41467_2021_26482_MOESM1_ESM.pdf]

Supplementary Information for

Magnetism-induced topological transition in $EuAs_3$

Erjian Cheng[1,*], Wei Xia[2,3,*], Xianbiao Shi[4,5,*], Hongwei Fang[2,6,*], Chengwei Wang[2,7], Chuanying Xi[8], Shaowen Xu[9], Darren C. Peets[10,11], Linshu Wang[1], Hao Su[2], Li Pi[8], Wei Ren[9], Xia Wang[2], Na Yu[2], Yulin Chen[2,3,12], Weiwei Zhao[4,5], Zhongkai Liu[2,3,#], Yanfeng Guo[2,¶], and Shiyan Li[1,13,14,§]

[1]*State Key Laboratory of Surface Physics, Department of Physics, and Laboratory of Advanced Materials, Fudan University, Shanghai 200433, China*

[2]*School of Physical Science and Technology, ShanghaiTech University, Shanghai 200031, China*

[3]*ShanghaiTech Laboratory for Topological Physics, Shanghai 201210, China*

[4]*State Key Laboratory of Advanced Welding & Joining and Flexible Printed Electronics Technology Center, Harbin Institute of Technology, Shenzhen 518055, China*

[5]*Key Laboratory of Micro-systems and Micro-structures Manufacturing of Ministry of Education, Harbin Institute of Technology, Harbin 150001, China*

[6]*University of Chinese Academy of Sciences, Beijing 100049, China*

[7]*State Key Laboratory of Functional Material for Informatics, Shanghai Institute of Microsystem and Information Technology, Chinese Academy of Sciences, Shanghai 200050, China*

[8]*Anhui Province Key Laboratory of Condensed Matter Physics at Extreme Conditions, High Magnetic Field Laboratory of the Chinese Academy of Sciences, Hefei, Anhui 230031, China*

[9]*Department of Physics, Shanghai University, Shanghai 200444, China*

[10]*Ningbo Institute of Materials Technology and Engineering, Chinese Academy of Sciences, Ningbo, Zhejiang 315201, China*

[11]*Institute for Solid State and Materials Physics, Technical University of Dresden, 01062 Dresden, Germany*

[12]*Department of Physics, University of Oxford, Oxford, OX1 3PU, United Kingdom*

[13]*Collaborative Innovation Center of Advanced Microstructures, Nanjing 210093, China*

[14]*Shanghai Research Center for Quantum Sciences, Shanghai 201315, China*

Supplementary Note 1: X-ray diffraction and thermodynamic measurements

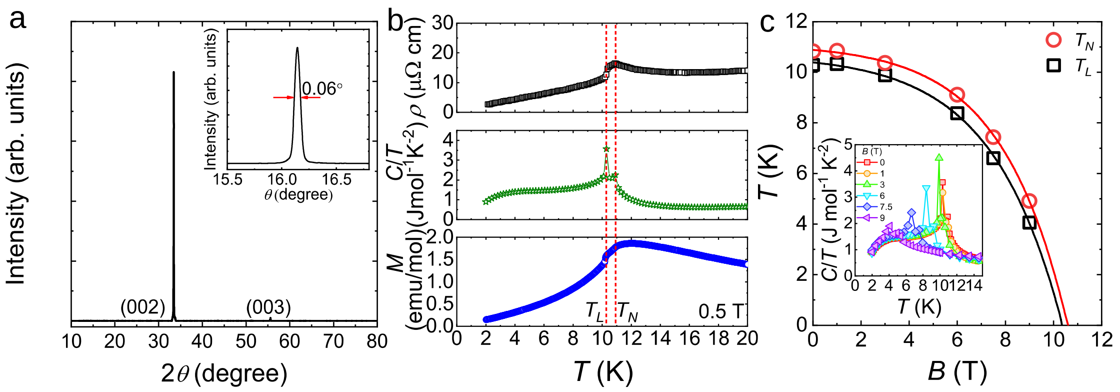

Supplementary Figure 1 | Basic physical properties of the as-grown $EuAs_3$ single crystals. a X-ray diffraction pattern from the largest surface of a $EuAs_3$ single crystal. The inset shows the rocking curve of the (002) peak. **b** Antiferromagnetic (T_N) and lock-in (T_L) transitions demonstrated by resistivity, specific heat and magnetic susceptibility. **c** Specific heat measurements under magnetic fields applied along the *c* axis.

Supplementary Fig. 1(a) shows the powder x-ray diffraction (XRD) pattern from the largest surface of a $EuAs_3$ single crystal. The inset shows the x-ray rocking curve of the (002) peak, the full width at half maximum (FWHM) of which is 0.06°, indicative of the high quality of our single crystals. Supplementary Fig. 1(b) shows the verification of the magnetic transitions by resistivity, specific heat and magnetic susceptibility, evidencing the antiferromagnetic (T_N) and lock-in (T_L) transitions at 11 and 10.3 K, respectively, consistent with previous reports[1]. Inset to Supplementary Fig. 1(c) displays specific heat measurements under magnetic fields. With increasing field, both T_N and T_L decrease. By extrapolating the data to zero temperature, as plotted in Supplementary Fig. 1(c), T_N and T_L will disappear at ~ 10.6 and ~ 10.4 T, respectively. To avoid any influence from the metamagnetic transition and investigate the fully spin-polarized state, we collected our quantum oscillation data above 11.0 T (Figs. 3(b) and 3(e) in the main text).

Supplementary Note 2: Angular-resolved magnetoresistance (AMR) in low magnetic field

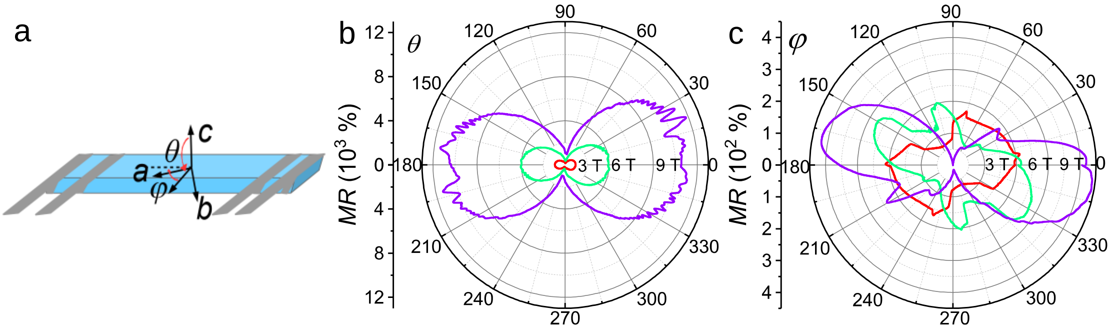

Supplementary Figure 2 | Angular-resolved magnetoresistance (AMR) of $EuAs_3$ single crystal at 2 K. a Schematic illustration of the experimental geometry and the angles θ and φ, and polar plots of the AMR for (**b**) θ and (**c**) φ at several magnetic fields.

Supplementary Fig. 2(a) illustrates the experimental geometry for our AMR measurements. For $\theta = 0°$ and 90°, the magnetic field is parallel to the *c* axis and the [110] direction, respectively. For $\varphi = 0°$, the magnetic field is parallel to the [110] direction in the *ab* plane, i.e., perpendicular to the electric current *I*. For $\varphi = 90°$, the magnetic field is parallel to the electric current *I*. Polar plots of the AMR for θ and φ at several magnetic fields are displayed in Supplementary Fig. 2(b) and 2(c), respectively, showing typical twofold anisotropy. Compared with θ, the AMR for φ is much more complicated, which is ascribed to the field-induced magnetic transitions[1]. For $\varphi \sim 90°$, i.e., magnetic field parallel to electric current *I*, a negative MR is observed. According to Supplementary Fig. 2(b) and 2(c), the scenario that external magnetic field suppresses the inelastic magnetic scattering from local moments or magnetic impurities and then induces a negative MR along all directions can be excluded.

Supplementary Note 3: Band structure in the spin-polarized state

The GGA+U (U = 5 eV) band structures of the spin-up and spin-down electrons,

shown in Supplementary Fig. 3(a) and 3(b), exhibit topological semimetal states with band crossings near the Fermi level. For both spin-up and spin-down channels, the band

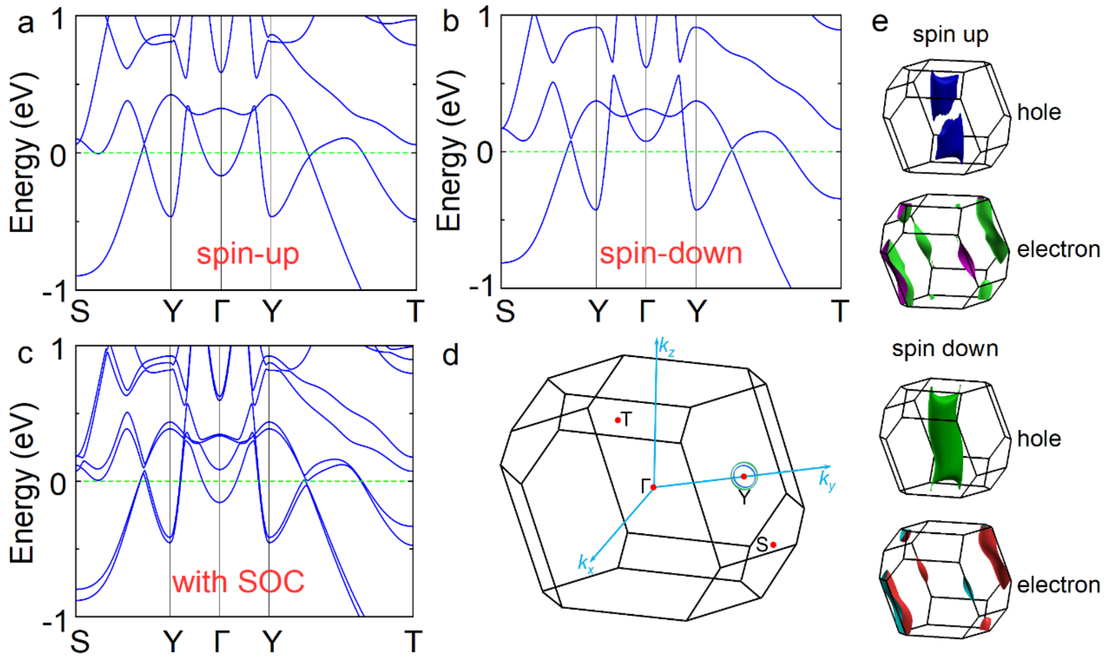

Supplementary Figure 3 | Topological nodal-line structure in the spin-polarized state of $EuAs_3$. Band calculations for the spin-polarized state of $EuAs_3$: (**a**) spin-up, (**b**) spin-down and (**c**) spin-orbit coupling (SOC)-involved band structures. **d** The bulk Brillouin zone of spin-polarized $EuAs_3$. The green and blue loops surrounding the *Y* point represent the nodal-line structure for the spin-up and spin-down channels, respectively. **e** Fermi surfaces of spin-polarized $EuAs_3$.

crossings persist along a closed path around the *Y* point in the BZ, showing a double nodal-line structure, as sketched in Supplementary Fig. 3(d). When spin-orbit coupling (SOC) is considered, the band crossings will be fully gapped because of the band hybridization, as shown in Supplementary Fig. 3(c). Supplementary Fig. 3(e) shows the Fermi surfaces of the spin-polarized $EuAs_3$ in presence of SOC. There are four main Fermi surface sheets—two hole sheets and two electron sheets. All these Fermi surfaces clearly display three-dimensional (3D) character.

Supplementary Note 4: Band structure in the paramagnetic state

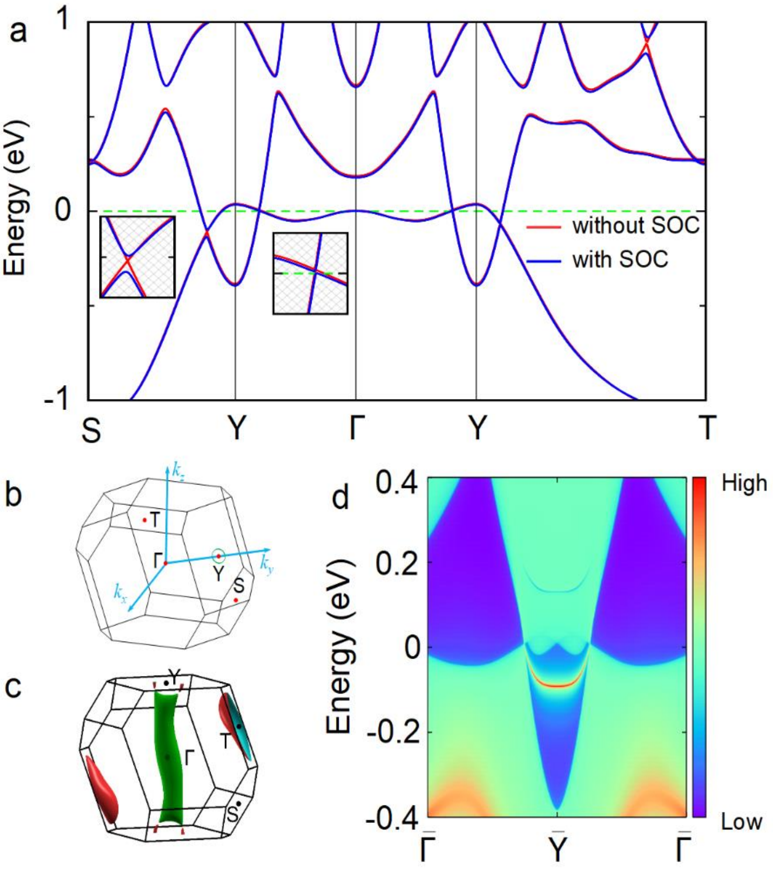

Supplementary Figure 4 | Topological nodal-line structure in the paramagnetic state of $EuAs_3$. a Band structure of $EuAs_3$. **b** The bulk Brillouin zone of the paramagnetic $EuAs_3$. A single nodal loop surrounding the *Y* point can be seen. **c** Fermi surface of paramagnetic $EuAs_3$. **d** The surface states of paramagnetic $EuAs_3$ on the projected surface perpendicular to the k_z axis.

In order to simulate the paramagnetic phase of $EuAs_3$, we treated the 4*f* electrons on Eu as core electrons. In the absence of SOC, the overall band profiles near the Fermi level, shown in Supplementary Fig. 4(a), share many key characteristics with those of $SrAs_3$ [2,3], making $EuAs_3$ a topological nodal-line semimetal (TNLS) in the paramagnetic state. Our band structure calculations indicate a nodal loop around the *Y* point in the BZ. When SOC is taken into consideration, gaps will be opened along the nodal line, and $EuAs_3$ becomes a small-gap insulator defined on curved Fermi levels.

The calculated surface states in Supplementary Fig. 4(d) illustrate a drumhead-like surface band, confirming further the TNLS character of paramagnetic $EuAs_3$. The Fermi surface in the paramagnetic state is calculated and displayed in Supplementary Fig. 4(c), and consists of two large sheets around the *Γ* and *T* points, and one small sheet around the *Y* point. The Fermi surface in the paramagnetic phase of $EuAs_3$ is very similar in shape to that of $SrAs_3$ [3], indicating that the nodal-line structure is quite robust, so direct verification of the nodal-line structure in the paramagnetic state by ARPES would serve as strong evidence for the existence of the nodal-line structure in the spin-polarized state.

Supplementary Note 5: ARPES results of Sample2 in the paramagnetic state

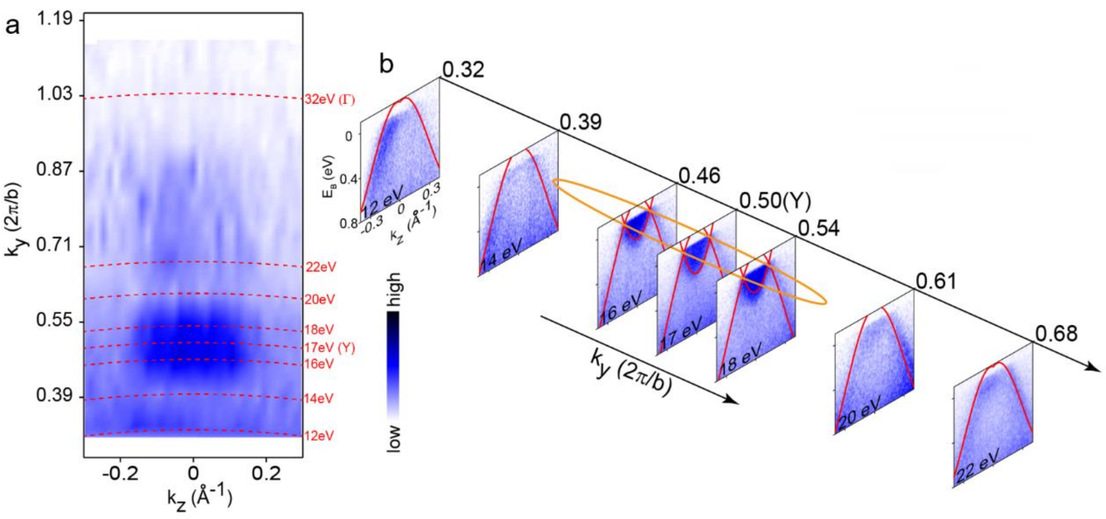

Supplementary Figure 5 | Verification of topological nodal-line structure by ARPES measurements at 18 K in the paramagnetic state of $EuAs_3$. a Photon-energy-dependent plot of photoemission intensities at the Fermi surface along the k_z direction. The red dotted lines denote the k_y momentum locations probed by different photon energies. **b** The dispersions along the k_z direction probed by different photon energies corresponding to different k_y. The calculated electronic structure is superimposed as red curves. The orange ellipse illustrates the topologically nontrivial

nodal loop.

We also re-measure the electronic structure of another $EuAs_3$ single crystal at 18 K within the vertical plane of the (010) cleaved surface, as shown in Supplementary Fig. 5. From the intensity plot of the Fermi surface in the k_y-k_z plane (Supplementary Fig. 5(a)), the pocket centered at the *Y* point (17 eV) can be easily identified, and two nodes arising from the crossing of the electronlike and holelike bands can be also observed in Supplementary Fig. 5(b), which agrees with the band calculations (red lines). For ARPES cuts away from the *Y* point (Supplementary Fig. 5(b)), the band-crossing area shrinks gradually and finally disappears. This is highlighted by an orange ellipse, and represents the nodal loop predicted by our band structure calculations.

Supplementary Note 6: Possible ferromagnetic fluctuations

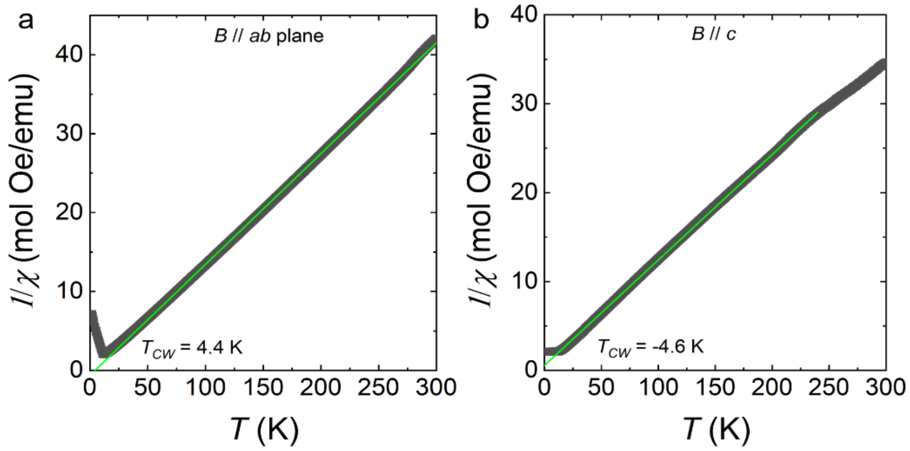

Supplementary Figure 6 | Temperature dependence of the inverse susceptibility of $EuAs_3$ single crystal. Magnetic fields were applied (**a**) in the *ab* plane, and (**b**) parallel to the *c* axis.

To check whether ferromagnetic correlations exist in the paramagnetic state, the Curie-Weiss temperature T_{CW} is extracted by fitting the temperature dependence of the inverse susceptibility to the Curie-Weiss law, as shown in Supplementary Fig. 6. A positive value of 4.4 K is extracted for magnetic fields applied in the *ab* plane,

evidencing possible ferromagnetic fluctuations.

Supplementary Note 7: Anomaly in resistivity below 3 K

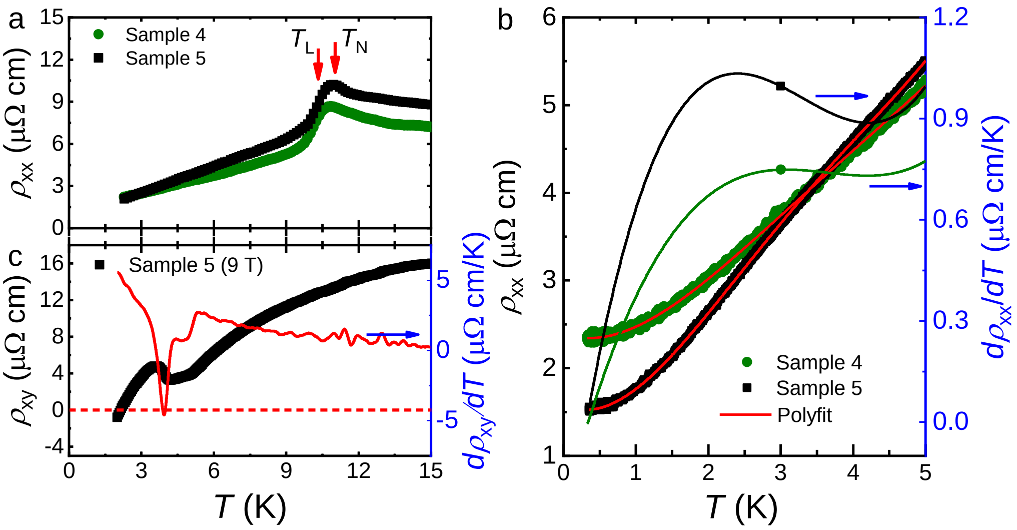

Supplementary Figure 7 | Low-temperature longitudinal and transverse resistivity of $EuAs_3$ single crystals. a The low-temperature longitudinal resistivity of two $EuAs_3$ single crystals. Two vertical arrows represent the temperatures where incommensurate antiferromagnetic (T_N) and incommensurate-to-commensurate lock-in (T_L) transitions take place. **b** The low-temperature longitudinal resistivity of these two single crystals measured in a ^{3}He cryostat. In order to avoid the influence of noise, we fit the data by using a polynomial to reproduce the experimental data first (the red solid lines). The olive and black lines are the derivative of the simulated data (the red solid lines) for Sample 4 and Sample 5, respectively. **c** The transverse Hall resistivity of $EuAs_3$ single crystal (Sample 5) at 9 T. The solid line represents the derivative of the data. Magnetic filed is applied along [110] direction.

Supplementary Fig. 7(a) shows the low-temperature resistivity of two more samples (Sample 4 and Sample 5), and the antiferromagnetic and incommensurate-to-commensurate lock-in transitions take place at 11 K and 10.3 K, respectively. As displayed in the inset of Fig. 2(a) in the main text, we didn't observe any distinct anomalies below 2.5 K. To check this, the low-temperature resistivity measurements

from 0.3 to 5 K for Sample 4 and Sample 5 have been performed, as shown in Supplementary Fig. 8(b), and there is no any distinct anomaly below 3 K. Considering that the variation in resistivity may be very weak and the weak signal may be covered by noise, we use a polynomial to reproduce the experimental data (the red solid lines in Supplementary Fig. 7(b)), and obtain the derivative from the simulated data. Surprisingly, a broad peak locating at ~2.3 K has been found in both Sample 4 and Sample 5. The peak in derivative suggests the change of slope in resistivity around 2.3 K, which maybe is relevant to the temperature-induced Lifshitz transition in the antiferromagnetic state.

For the high field range, we conducted the Hall coefficient measurements at 9 T, shown in Supplementary Fig. 7(c). As one can see, no clear anomalies can be observed around T_L and T_N, while a small peak at ~3.6 K arises and Hall coefficient changes its sign from positive to negative at ~2.3 K.

Supplementary Note 8: Quantum oscillations in the Hall resistivity

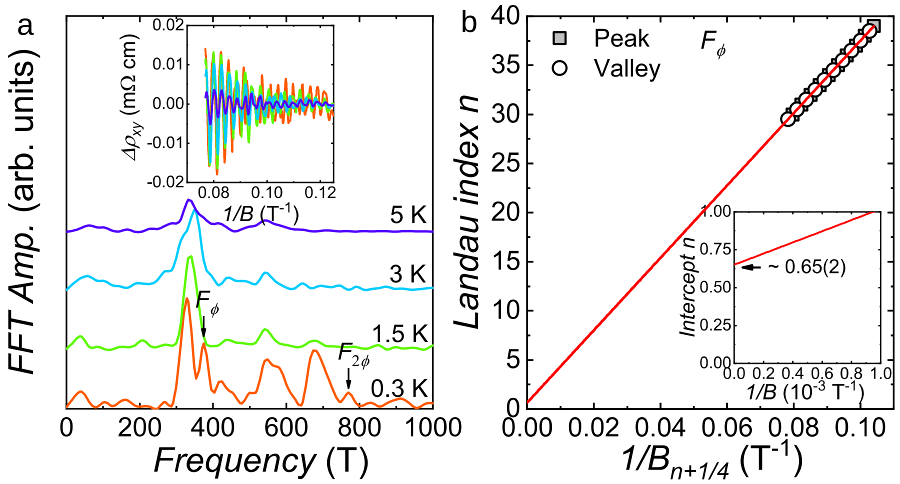

Supplementary Figure 8 | The analysis of Hall resistivity oscillations of $EuAs_3$ single crystal. a FFT results based on Hall resistivity oscillations at several temperatures. The inset shows the oscillatory component of ρ_{xy} as a function of $1/B$. A new frequency denoted as F_ϕ identifies a new ϕ band. **b** Landau index n as a function of $1/B_{n+1/4}$ in consideration of a 1/4 phase shift[4]. The red line represents a linear fit. The

inset shows the extrapolation of $1/B$ to zero.

To check the temperature-induced Lifshitz transition, we analyze the oscillatory component ($\Delta\rho_{xy}$) (displayed in the inset to Supplementary Fig. 8(a)) via FFT, and a new band (denoted as ϕ) with oscillation frequency of 374 T has been identified, demonstrating the Lifshitz transition. We then check the topology of the φ band, as plotted in Supplementary Fig. 8(b). We assign integer indices to the $\Delta\rho_{xy}$ peak positions in $1/B_{n+1/4}$ and half integer indices to the $\Delta\rho_{xy}$ valleys, where n+1/4 means the 1/4 phase shift in Hall resistivity compared with longitudinal resistivity[4]. The intercept of 0.65(2) falls in the range between 3/8 and 5/8, as shown in the inset to Supplementary Fig. 8(b), suggesting the possibly trivial topology of the ϕ band.

Supplementary Note 9: Exclusion of the open-orbit effect for XMR

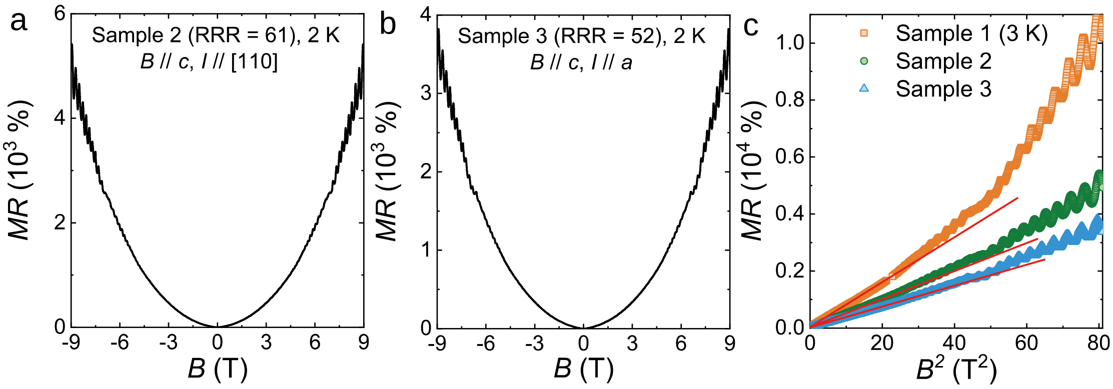

Supplementary Figure 9 | Magnetoresistance (MR) measurements of $EuAs_3$ single crystal at 2 K with different current direction. a For Sample 2, magnetic field is applied along the c axis, and electric current I along the [110] direction. **b** For Sample 3, magnetic field is applied along the c axis, and electric current I along the a axis. **c** B^2 as a function of MR for three different samples. The data for Sample 1 is taken from that in Fig. 2(b). With changing the direction of electric current, the unsaturated XMR persists, excluding the open-orbit effect.

To exclude the open-orbit effect which may also induce the XMR in $EuAs_3$, we

performed the MR measurements with electric current I applied along different crystallographic axis, as shown in Supplementary Fig. 9. Supplementary Fig. 9(a) shows the MR of Sample 2 with magnetic field parallel to c axis and electric current applied along the [110] direction, which is the same as Sample 1 (Fig. 2(b)). Compared with Sample 1, the MR of Sample 2 with RRR ~61 is smaller than that of Sample 1, indicating that the quality of single crystal may have a great effect on the magnitude of MR. For Sample 3 with smaller RRR ~52, the MR is smaller than those of Sample 1 and Sample 2. For comparison, we plot the B_2 dependence of MR for these three samples, as shown in Supplementary Fig. 9(c), which displays similar behavior in spite of the magnitude of MR (The difference may come from the different quality of single crystals.). According to the open-orbit effect, the unsaturated XMR is only observed for current along the open orbits, which means that changing the current direction will lead to a saturated MR[5]. Therefore, the unsaturated XMR with different current direction in $EuAs_3$ suggests that the XMR resulting from the open-orbit effect can be excluded.

Supplementary References

1. Bauhofer, W. and McEwer, K. A. Anisotropic magnetoresistance of the semimetallic antiferromagnet $EuAs_3$. *Phys. Rev. B* **43**, 13450-13455 (1991).
2. Xu, Q. *et al.* Topological nodal line semimetals in CaP_3 family of materials. *Phys. Rev. B* **95**, 045136 (2017).
3. Li, S. C. *et al.* Evidence for a Dirac nodal-line semimetal in $SrAs_3$. *Sci. Bull.* **63**, 535-541 (2018).
4. He, L. P. *et al.* Quantum Transport Evidence for the Three-Dimensional Dirac Semimetal Phase in Cd_3As_2. *Phys. Rev. Lett.* **113**, 246402 (2014).
5. Zhang, S. N. *et al.* Magnetoresistance from Fermi surface topology. *Phys. Rev. B* **99**, 035142 (2019).
